# Editorial

**Published:** 1899-06

**Authors:** 


### HOMŒOPATHIC PHYSICIAN,

A MONTHLY JOURNAL OF

HOMŒOPATHIC MATERIA MEDICA AND CLINICAL MEDICINE.

If our school ever gives up the strict inductive method of Hahnemann, we are lost, and deserve only to be mentioned as a caricature in the history of medicine."—CONSTANTINE HERING.

Vol. XIX. JUNE, 1899. No. 6.

### EDITORIAL.

THE USE OF ANTI-TOXIN as preventive of diphtheria is still being strongly advocated in many quarters, and it is probable that it will not be very long before the inoculation of this new remedy will become legally obligatory, unless the efforts now being made to repeal compulsory vaccination laws shall be successful. If this latter object be attained, it will, of course, have a discouraging effect upon the efforts to make inoculation of anti-toxin universal.

Physicians of every point of view on this subject should study more closely just what it means to inject these unknown and virulent fluids into the system of a human being.

For this purpose the most exhaustive examination, especially by means of chemical analysis, must be made.

We are all wanting in understanding of the subject, because we have not studied it from this standpoint.

Yet some idea of it may be gained by referring back to the phenomena of fermentation.

A ferment is a plant which, falling into an organic fluid, takes from that fluid by decomposition what is needed for its own sustenance and leaves a residue which rearranges itself into new compounds, just as when the keystone is taken from the arch, the arch falls into ruins of more or less fantastic new shapes.

When the yeast plant falls into grape juice, it decomposes the juice in order to find food for itself. If an animal feed on this same grape juice, it takes the whole fluid unaltered into its economy and whatever decomposition occur is within the confines of its system. This is the essential characteristic of animal digestion. But the yeast plant in feeding brings about the decomposition outside of its own economy, abstracting just as much of the carbon and hydrogen atoms as are needful for its sustenance, and, rejecting the rest, which falls into new shapes like the broken arch. These new shapes being alcohol, carbonic acid water and various others which give the bouquet. When the diphtheria germ is implanted into the juices of the horse or other animal it feeds upon these juices, abstracting as much of the carbon, hydrogen, and nitrogen elements as are needed for its own use, just as the yeast plant does in the grape juice. The remainder reform themselves into various compounds of the most unexpected character. As these processes take place in *animal* fluids, which are more complex than vegetable substances, the products are more complicated in their composition. They are very changeable and are tremendously poisonous.

Animal substances generally are built up of various combinations of carbon, hydrogen, and nitrogen, in certain complex compounds. These compounds are united with each other to make the resulting products still more complex, and these combinations seem to be very loosely held together, so that they are liable to extensive changes from seemingly slight causes. Of these causes the principal ones are the various microscopic plants, such as molds and other fungi, and bacteria. When these plants produce their changes, the results of the decomposition are very varied and for the most part unknown, while all have high toxic power. We are all familiar with the dangers from putrefaction of animal bodies. All students of medicine know well the dangers from dissection wounds. The disasters from such accidents fall directly under the remarks here made. The products which produce the terrible illness which follows a prick of the finger while dissecting a dead human being or animal, can be isolated and exhibited to the eye and their qualities further demonstrated by chemistry and pathogenesis.

The inoculation of anti-toxin or of vaccine is not essentially different from the dissection wound. The results are liable to be the same. Yet notwithstanding this scientific parallel, we medical men do not hesitate to practice inoculation or vaccination with rarely a misgiving as to the outcome, while we fly from the dissection wound in terror.

Therefore, if we persist in using such agents upon human beings, it is our duty to find out their exact chemical composition, isolate the active principles which give them virulence and examine their pathogenic spheres of action that we may know exactly what we dare do with them.

As it is we administer them blindly and it is only a matter of chance whether they produce instant death (as has occurred more than once) or prolonged and unnecessary sickness or are actually curative.

With this uncertainty they represent the very worst form of empiricism. Homœopathy is a continual protest against that, but as numbers of men in our school nevertheless continue to use anti-toxin, they ought at least to make themselves familiar with the pecularities of the remedies they use.

